# PFOS Disrupts Oocyte Maturation and Early Embryonic Development via Ovarian FOXK1 O‐GlcNAcylation in Mice

**DOI:** 10.1002/advs.202514857

**Published:** 2025-12-12

**Authors:** Shuwen Han, Qin Yuan, Zhu Wu, Yaohui Fang, Hong Qian, Jiale Zhu, Yuchen Zhang, Ke Deng, Liangliang Su, Haibo Xu, Haotian Shu, Yiming Gong, Qiaoqiao Xu, Guizhen Du, Di Wu, Yun Fan, Chuncheng Lu

**Affiliations:** ^1^ State Key Laboratory of Reproductive Medicine Center for Global Health School of Public Health Nanjing Medical University Nanjing 211166 China; ^2^ Key Laboratory of Modern Toxicology of Ministry of Education, School of Public Health Nanjing Medical University Nanjing 211166 China; ^3^ Department of Microbes and Infection School of Public Health Nanjing Medical University Nanjing 211166 China

**Keywords:** PFOS, O‐GlcNAcylation, oocyte maturation, early embryonic development, progesterone

## Abstract

Perfluorooctane sulfonate (PFOS) is of great concern due to its accumulation in living organisms and reproductive toxicity. Although prior studies indicate that PFOS exposure causes female reproductive disorders, the underlying mechanism remains obscure. This study investigates the molecular mechanisms underlying PFOS‐induced female reproductive toxicity at human‐relevant exposure levels. These results demonstrate that PFOS exposure (0.2 and 20 µm) significantly reduces polar body extrusion (PBE) and delays germinal vesicle breakdown (GVBD) in oocytes. Additionally, PFOS exposure (1 mg kg^−1^ day^−1^) decreases the proportion of two‐cell embryos and reduces progesterone (P4) levels. Elevated O‐GlcNAcylation levels are observed in both ovaries and granulosa cells (GCs) under PFOS treatment. Proteomic profiling of protein O‐GlcNAcylation identifies that the O‐GlcNAcylation of forkhead box k1 (FOXK1) at threonine (Thr) 573 cite involved in ovarian steroidogenesis. Mechanistically, co‐immunoprecipitation (Co‐IP) combined with LC‐MS/MS analysis reveals a physical interaction between FOXK1 and pescadillo ribosomal biogenesis factor 1 (PES1). Increased O‐GlcNAcylation of FOXK1 at Thr573 inhibits the ubiquitination‐mediated degradation of PES1, leading to elevated PES1 expression. Furthermore, PES1 promotes aldo‐keto reductase family 1, member C18 (AKR1C18) to reduce P4 levels, ultimately disrupting oocyte maturation and early embryonic development. Overall, this study provides valuable insights into the role of protein post‐translational modifications in oocyte maturation and embryonic development under PFOS exposure.

## Graphical abstract

Perfluorooctane sulfonate (PFOS) exposure disrupts oocyte maturation and early embryonic development. This study elucidates the mechanism by which enhanced O‐GlcNAcylation of FOXK1 underlies the PFOS‐induced reduction of progesterone levels in granulosa cells and the disturbance of follicular microenvironment.

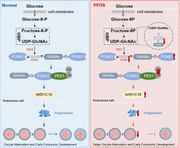

## 1 Introduction

Perfluorooctane sulfonate (PFOS), a legacy polyfluoroalkyl and perfluoroalkyl substance (PFAS), has been extensively utilized in firefighting foams, textiles, and food packaging.^[^ ^1^ ^]^ Despite its inclusion in the Stockholm Convention for restriction in 2009, PFOS remains ubiquitously detected in environmental and human biological samples.^[^ ^2^ ^]^ Studies have reported widespread detection of PFOS in prenatal and postnatal blood samples and umbilical cord blood,^[^ ^3^ ^]^ suggesting that PFOS exposure may contribute to reproductive disorders and adverse pregnancy outcomes. Toxicological studies confirmed that PFOS can reduce fertilization and implantation rates, disrupt reproductive hormone levels, and result in preterm birth and low birth weight.^[^ ^4^, ^5^, ^6^, ^7^, ^8^ ^]^

Abnormalities in oocyte maturation and early embryonic development are major reproductive phenotypes associated with reduced female fertility.^[^ ^9^ ^]^ Oocyte maturation, characterized by meiotic resumption and cytoplasmic maturation, is critical for determining fertilization potential and embryo quality.^[^ ^10^, ^11^ ^]^ Previous studies found that PFOS exposure disrupted meiotic progression in mouse oocytes and reduced the number of metaphase II (MII)‐stage oocytes.^[^ ^12^, ^13^ ^]^ Long‐chain PFAS affected the hypothalamic‐pituitary‐ovarian (HPO) axis by interfering with follicle‐stimulating hormone (FSH)/luteinizing hormone (LH) signaling pathway, resulted in abnormal hormone secretion and consequent disruption of oocyte development.^[^ ^14^ ^]^

Granulosa cells (GCs) constitute the largest cellular population in ovarian follicles and play essential roles in supporting oocyte development.^[^ ^15^ ^]^ FSH primarily stimulates granulosa cell proliferation and the aromatization of androgens into estradiol (E2).^[^ ^16^ ^]^ FSH and LH drive granulosa cell proliferation and steroidogenesis (E2 and progesterone (P4)).^[^ ^17^ ^]^ P4 mediates the resumption of meiosis and coordinates cytoplasmic differentiation, thereby conferring fertilization competence to oocytes and supporting early embryogenesis.^[^ ^18^ ^]^ PFOS exposure has been demonstrated to suppress steroidogenesis in GCs through multiple pathways, including the suppression of key steroidogenic enzyme via reduced histone acetylation and the impairment of mitochondrial function.^[^ ^19^, ^20^ ^]^ However, the precise intracellular signaling mechanisms remain poorly understood.

Protein O‐linked beta‐N‐acetylglucosaminylation (O‐GlcNAcylation), a dynamic post‐translational modification, acts as a vital nutrient and stress sensor, which significantly alters protein structure and stability.^[^ ^21^, ^22^ ^]^ O‐GlcNAcylation plays a critical role in oocyte maturation and embryonic development by regulating key cellular events.^[^ ^23^, ^24^, ^25^ ^]^ Our previous research found that PFOS exposure interferes with the hexosamine biosynthesis pathway (HBP)‐mediated alterations in O‐GlcNAcylation levels.^[^ ^26^ ^]^ Elevated O‐GlcNAcylation promoted the transcription of pro‐apoptotic genes, leading to GCs apoptosis.^[^ ^27^, ^28^ ^]^ However, whether O‐GlcNAcylation functioned in the steroid hormone synthesis of GCs under PFOS exposure remains poorly understood.

Here, we established a PFOS‐exposed mouse model to investigate the effects of human‐related exposure dose of PFOS on oocyte meiotic and early embryonic development. We observed that PFOS exposure delayed oocyte meiotic progression, impaired early embryonic development at the two‐cell stage, and reduced steroid hormone in a dose‐dependent manner. Mechanistically, using GCs and human embryonic kidney 293T (HEK‐293T cell) line models, we explored the mechanistic role of FOXK1 O‐GlcNAcylation‐mediated suppression of PES1 ubiquitination and degradation in the abnormal oocyte maturation and early embryonic development induced by PFOS. Overexpression of AKR1C18 increased progesterone catabolism. This study provides novel insights into the mechanisms underlying the reproductive toxicity of PFOS.

## 2 Results

### 2.1 PFOS Exposure Delayed Oocyte Meiotic Maturation and Early Embryonic Development in a Dose‐Dependent Manner

**Figure 1:**
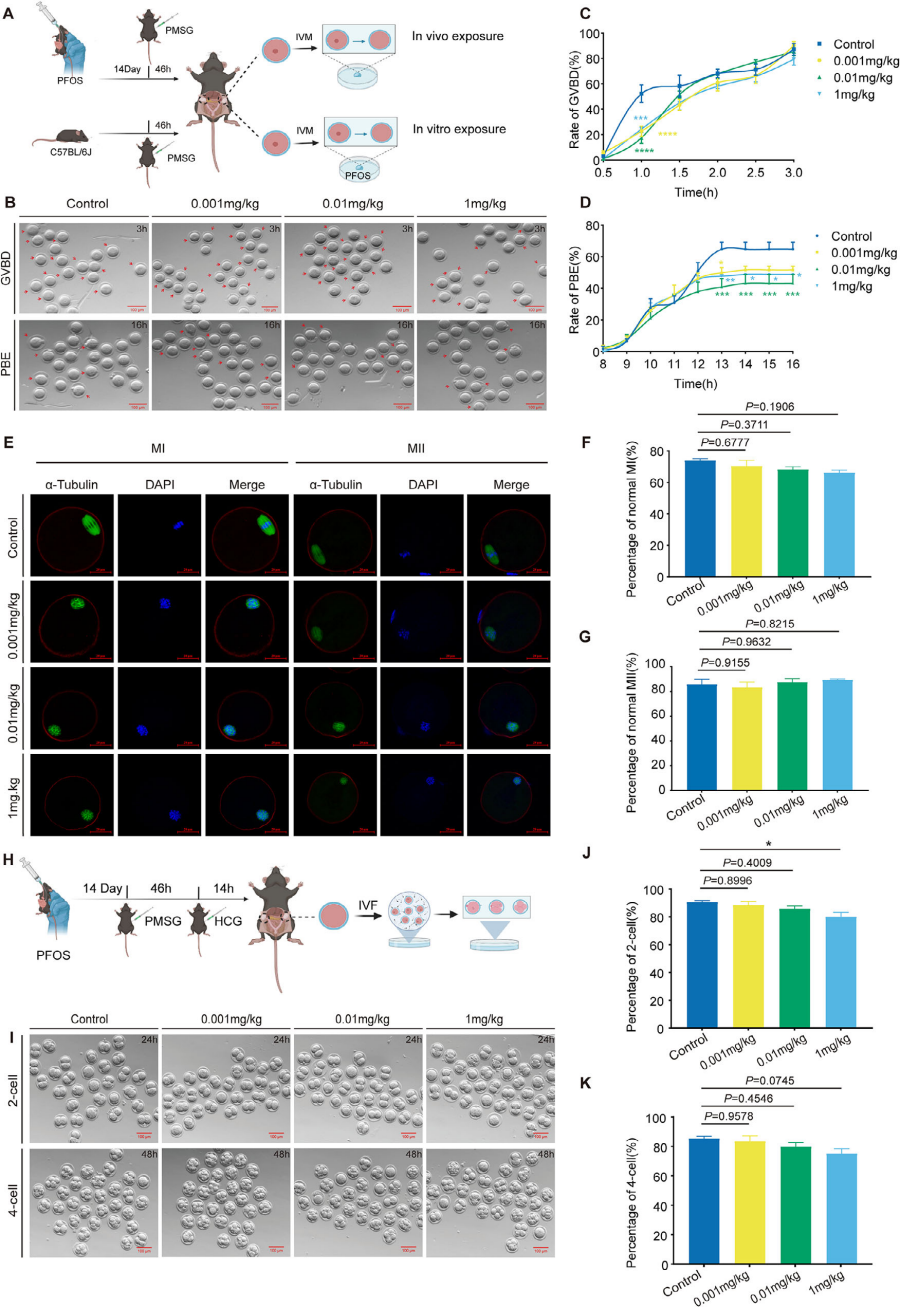
PFOS exposure delayed oocyte meiotic maturation and early embryonic development. A) Schematic of the in vivo and in vitro PFOS exposure models in mice (0.001, 0.01, and 1 mg kg^−1^ day^−1^). B) Representative images of GVBD (after 3 h in vitro maturation) and PBE (after 16 h in vitro maturation) in oocytes from PFOS‐exposed mice (Bar = 100 µm). C) GVBD rate in oocytes from PFOS‐exposed mice (Control, *n* = 69; 0.02 µm, *n* = 124; 0.2 µm, *n* = 97; 20 µm, *n* = 103). D) PBE rate in oocytes from PFOS‐exposed mice (Control, *n* = 69; 0.02 µm, *n* = 124; 0.2 µm, *n* = 97; 20 µm, *n* = 103). E) Immunofluorescence images of spindles and chromosomes in MI‐ and MII‐stage oocytes from PFOS‐exposed mice (after 16 h in vitro maturation; Bar = 20 µm). F) Proportion of MI oocytes with normal spindle/chromosome alignment (Control, *n* = 27; 0.02 µm, *n* = 54; 0.2 µm, *n* = 47; 20 µm, *n* = 53). G) Proportion of MII oocytes with normal spindle/chromosome alignment (Control, *n* = 26; 0.02 µm, *n* = 41; 0.2 µm, *n* = 32; 20 µM, *n* = 36). H) Pattern diagram of IVF. I) Representative pictures of oocytes in vitro fertilization of early embryos (two‐cell embryos, 24 h after fertilization; four‐cell embryos, 48 h after fertilization; Bar = 100 µm). J) The rate of development of two‐cell embryos (24 h after fertilization; Control, *n* = 297; 0.02 µm, *n* = 287; 0.2 µm, *n* = 374; 20 µm, *n* = 360). K) The rate of development of four‐cell embryos (48 h after fertilization; Control, *n* = 297; 0.02 µm, *n* = 287; 0.2 µm, *n* = 374; 20 µm, *n* = 360). Statistics: Data are represented as mean ± SEM. *P* values determined by one‐way ANOVA or two‐way ANOVA followed by Dunnett's or Tukey's multiple comparisons test. ^*^ *P* < 0.05; ^**^ *P* < 0.01; ^***^ *P* < 0.001; ^****^ *P* < 0.0001.

To explore the effect of PFOS exposure on oocyte maturation, we constructed mouse models of PFOS exposure both in vivo and in vitro (**Figure** **1**A). In vitro maturation (IVM) was applied to assess the oocyte germinal vesicle breakdown (GVBD) and first polar body exclusion (PBE) after a two‐week treatment of PFOS (0.001, 0.01, and 1 mg kg^−1^ day^−1^) in mouse oocytes (Figure 1B). PFOS exposure (0.001, 0.01, and 1 mg kg^−1^ day^−1^) caused the delay of GVBD in oocytes at the early stage, and the difference gradually decreased after 1.5 h (Figure 1C). Also, we observed that exposure to PFOS (0.001, 0.01, and 1 mg kg^−1^ day^−1^) significantly reduced rates of PBE in mouse oocytes (Figure 1D). As shown in Figure 1E–G, exposure to PFOS did not damage oocyte spindle and chromosomes in vivo.

Next, we obtained germinal vesicle oocytes from wild‐type mice and then treated them with PFOS (0.02, 0.2, and 20 µm) or carrier control (0.001% DMSO) in vitro (Figure 1A). After 20 µm PFOS treatment, the GVBD process was significantly delayed, and the rate of GVBD was decreased (Figure S1A, Supporting Information). We found significantly lower PBE rates in oocytes exposed to 0.2 and 20 µm PFOS compared to the controls (Figure S1B, Supporting Information). Consistent with the in vivo experiments, we did not find any abnormalities in the oocyte spindle or chromosomes (Figure S1C–E, Supporting Information). Taken together, our results suggested that PFOS exposure disrupted oocyte maturation in vivo and in vitro, but did not affect the meiosis of the oocyte.

Oocyte quality is a vital limiting factor for female successful fertilization. To investigate whether PFOS exposure affects the development of early embryos, in vitro fertilization (IVF) was conducted with MII stage oocytes after mice were exposed to PFOS (0.001, 0.01, and 1 mg kg^−1^ day^−1^) (Figure 1H). PFOS exposure decreased the developmental rate of two‐cell embryos in a dose‐dependent manner, with a statistically significant difference observed in the 1 mg kg^−1^ day^−1^ PFOS group (Figure 1I,J). The rate of development of four‐cell embryos, morula and blastula stages showed a similar downward trend and fell below 80% in the high‐dose group (Figure 1K; Figure S1F–G, Supporting Information). These results indicated that PFOS exposure led to early embryonic development retardation at the two‐cell stage in a dose‐dependent manner.

### 2.2 PFOS Exposure Delayed Oocyte Maturation and Early Embryonic Development via Reducing Steroid Hormone Levels

**Figure 2:**
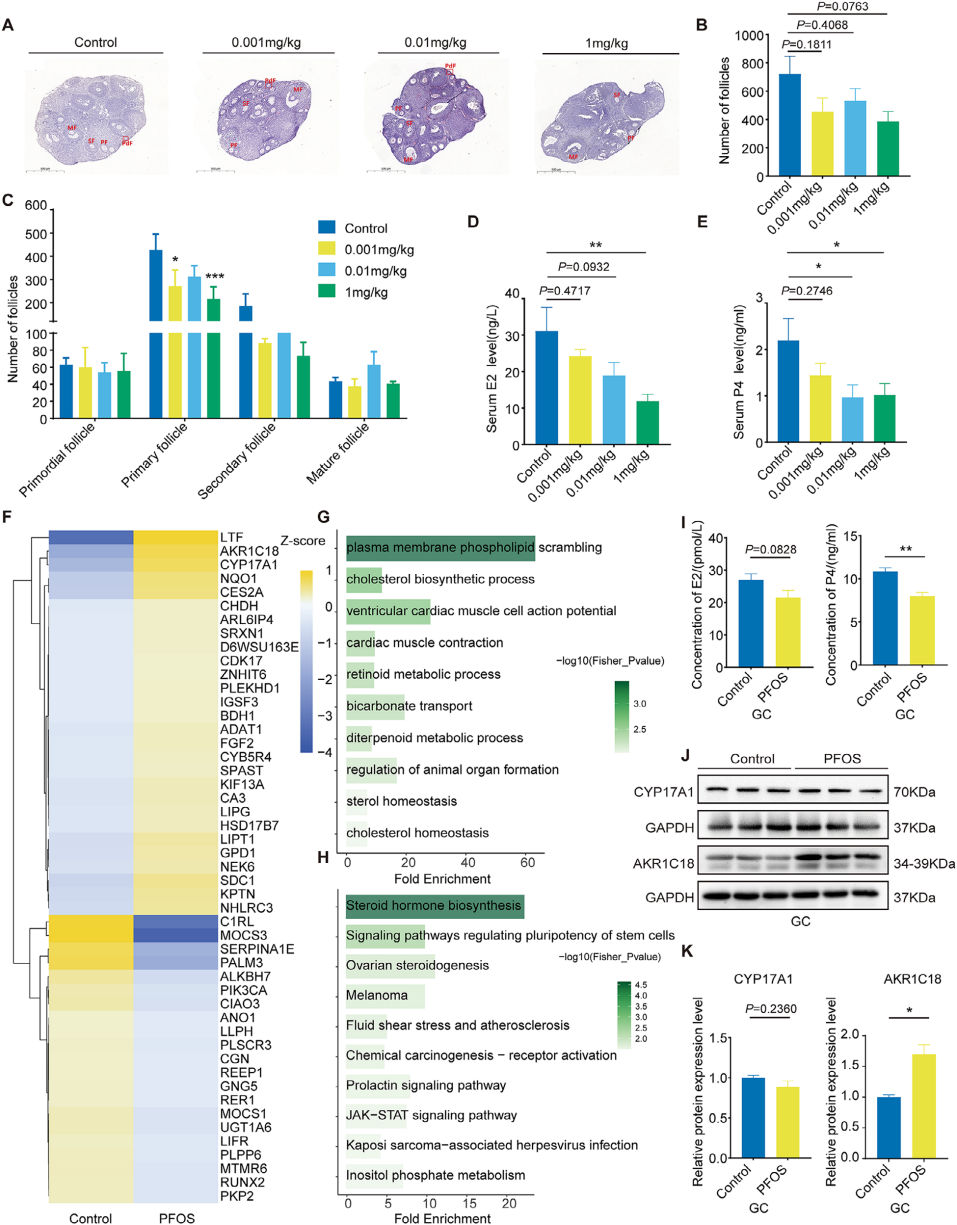
PFOS exposure delayed oocyte maturation and early embryonic development via reducing steroid hormone levels. A) Representative micrographs of H&E staining of ovaries from PFOS‐exposed mice (PdF: Primordial follicle; PF: Primary follicle; SF: Secondary follicles; MF: Mature follicle; Bar=500 µm). B) Total number of ovarian follicles (*n* = 4). C) Number of follicles at various stages (*n* = 4). D) Serum E2 levels (*n* = 8). E) Serum P4 levels (*n* = 11–12). F) Clustered heatmap of differentially expressed proteins. G) GO analysis of differentially expressed proteins in mouse ovaries exposed to PFOS. H) KEGG analysis of differentially expressed proteins in mouse ovaries exposed to PFOS. I) E2 and P4 levels in GCs exposed to 20 µm PFOS (*n* = 9). J) Western blot bands of protein of CYP17A1, AKR1C18 in GCs exposed to 20 µm PFOS (*n* = 3). K) Relative protein expression levels of CYP17A1 and AKR1C18 (*n* = 3). Statistics: Data are represented as mean ± SEM. *P* values were determined by one‐way ANOVA with Dunnett's or Tukey's multiple comparisons test and two‐tailed unpaired *t*‐tests. ^*^ *P* < 0.05; ^**^ *P* < 0.01.

The ovarian follicle provides a specialized microenvironment that supports oocyte maturation and female fertility.^[^ ^29^ ^]^ To assess the effects of PFOS exposure on follicular development, we performed H&E staining of the mouse ovarian serial sections and counted the number of follicles at various stages of follicle development (**Figure** **2**A). PFOS exposure showed a non‐significant trend toward reducing the total number of ovarian follicles. The largest reduction reached 53.5% of the control group (Figure 2B). The number of follicles at various stages showed that PFOS (1 mg kg^−1^) exposure resulted in a significant reduction in the number of primary follicles (Figure 2C), suggesting PFOS may interfere with follicular development and thus disrupt the microenvironment for oocyte growth and development.

Ovarian follicles are the fundamental structures that support oocyte development by producing steroid hormones,^[^ ^30^, ^31^ ^]^ so we investigated serum E2 and P4 levels in PFOS‐exposed mice. Compared with the control group, serum E2 levels gradually decreased in a dose‐dependent manner of PFOS treatment, and the difference was significant in the 1 mg kg^−1^ day^−1^ PFOS group (Figure 2D). In addition, exposed to PFOS (0.01 and 1 mg kg^−1^) led to a significant decrease of P4 levels (Figure 2E). These results indicate that PFOS may affect follicular development by interfering with ovarian steroid hormone levels.

To further explore the mechanisms that PFOS reduces steroid hormone to regulate oocyte and follicle development, we collected ovaries from the PFOS group (1 mg kg^−1^) and the control group for LC‐MS/MS analysis. We totally identified 49 differentially expressed proteins (DEPs) (Fold Change > 1.5, *P* < 0.05; Table S1, Supporting Information), of which 28 were up‐regulated (e.g., AKR1C18, CYP17A1, and HSD17B7) and 21 were down‐regulated (e.g., C1RL, MOCS3, and PIK3CA) (Figure 2F). Gene ontology (GO) enrichment and Kyoto Encyclopedia of genes and genomes (KEGG) analysis showed that these DEPs were significantly enriched in steroid hormone biosynthesis, ovarian steroidogenesis, and phosphatidylinositol signal system (Figure 2G,H).

GCs promote follicular development through E2 and P4 synthesis stimulated by FSH/LH, while their dysfunction impairs both sex hormone production and follicular development.^[^ ^32^, ^33^ ^]^ We chose the mouse ovarian GC line for subsequent in vitro experiments (Figure S2A, Supporting Information). A dose of 20 µm PFOS (equivalent to 1 mg kg^−1^ day^−1^ in mouse model in vivo), was selected for exposure. As shown in Figure 2I, the GC supernatant levels of E2 and P4 decreased, with a significant difference in P4 after PFOS treatment. Next, we assessed the protein levels of several key sterol hormone regulators (CYP11A1, 3β‐HSD, CYP17A1, and AKR1C18) in GCs. PFOS exposure did not alter the expression of CYP11A1, 3β‐HSD, or CYP17A1, but significantly elevated AKR1C18 (Figure 2J,K; Figure S2B–D, Supporting Information). Meanwhile, the protein level of estrogen receptor β (ERβ) was significantly downregulated, while the decrease trend was found in estrogen receptor α (ERα) with no significant difference (Figure S2E–G, Supporting Information).

### 2.3 PFOS Exposure Increased O‐GlcNAcylation by Reducing OGA to Inhibit Removing the GlcNAc Moiety

**Figure 3:**
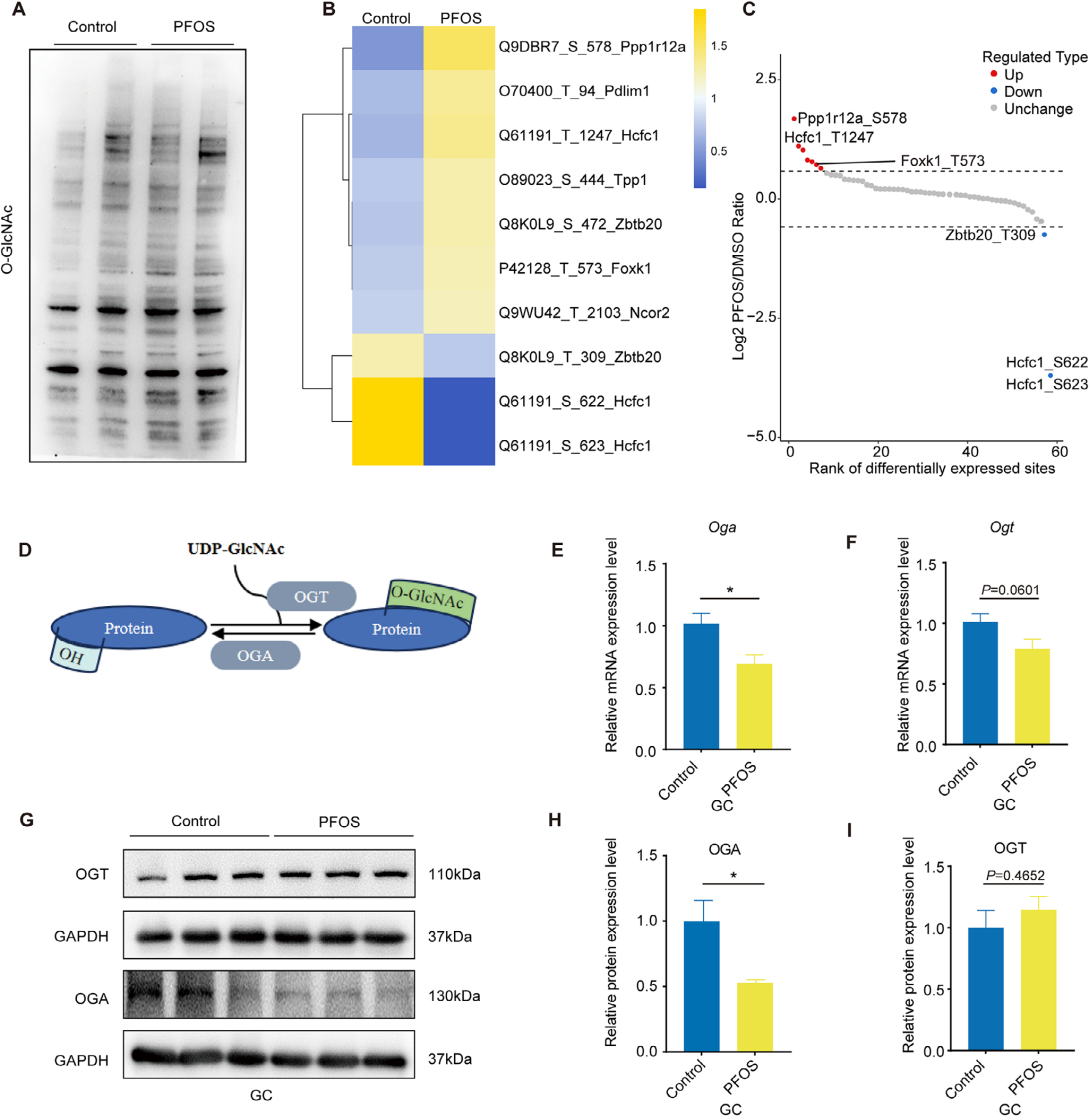
PFOS exposure increased O‐GlcNAcylation by reducing OGA to inhibit removing the GlcNAc moiety. A) Total O‐GlcNAcylation levels in ovaries from PFOS‐exposed mice (*n* = 2). B) Heat map of different O‐GlcNAcylation protein sites in PFOS‐exposed mice ovaries. C) Scatter plot of different O‐GlcNAcylation protein sites in PFOS‐exposed mice ovaries. D) Process of the occurrence of O‐GlcNAcylation. E,F) Gene expression levels of *Ogt* and *Oga* in GCs exposed to PFOS (*n* = 6). G–I) Relative protein expression level of OGT and OGA in GCs exposed to PFOS (*n* = 3). Statistics: Data are represented as mean ± SEM. *P* values were determined by two‐tailed unpaired *t*‐tests. ^*^ *P* < 0.05; ^**^ *P* < 0.01.

Our previous study showed that PFOS significantly affected HBP, and affected the level of its end product uridine diphosphate N‐acetylglucosamine (UDP‐GlcNAc). UDP‐GlcNAc is reported to act as a substrate for O‐GlcNAcylation and mediates O‐GlcNAcylation alteration.^[^ ^34^, ^35^ ^]^ We hypothesized that O‐GlcNAcylation played an important role in PFOS‐induced steroid hormone reduction in this study. To further explore the effects of PFOS exposure on O‐GlcNAcylation, we first assessed total O‐GlcNAcylation levels after PFOS exposure (1 mg kg^−1^ and 20 µm). Results showed a marked increase in total O‐GlcNAcylation levels in both ovaries and GCs in the PFOS‐treated group (**Figure** **3**A; Figure S3A, Supporting Information). Next, we collected ovaries from the PFOS group (1 mg kg^−1^) and the control group for LC‐MS/MS analysis of O‐GlcNAcylation modification characterization. In total, seven differentially modified proteins (FOXK1, HCFC1, TPP1, ZBTB20, NCOR2, PDLIM1, and PPP1R12A) and ten differentially modified sites were identified after PFOS exposure (Fold change > 1.5, *P* < 0.05, Figure 3B,C; Table S2, Supporting Information).

Protein O‐GlcNAcylation is affected by O‐GlcNAcase (OGA), O‐GlcNAc transferase (OGT) and the substrate UDP‐GlcNAc (Figure 3D; Figure S3B, Supporting Information).^[^ ^36^ ^]^ To decipher the reasons for PFOS‐induced O‐GlcNAcylation modification alterations, we incubated GCs with exogenous addition of 2 mm UDP‐GlcNAc for 48 h. The results showed that UDP‐GlcNAc treatment increased the overall O‐GlcNAcylation level of GCs in the control group, while decreased in the PFOS‐exposed group when compared to no UDP‐GlcNAc treatment, respectively (Figure S3C,D, Supporting Information). We next assessed the effect of PFOS exposure on the expression levels of key enzyme genes (such as *Glut4*, *Hk1*, *Gpi*, *Gfpt1*, *Pgm3*, *Gna1*, and *Uap1*) during UDP‐GlcNAc generation, and RT‐qPCR results showed no significant changes in their mRNA levels (Figure S3E, Supporting Information). We also found that PFOS exposure significantly reduced OGA protein level and mRNA level while OGT expression remained unchanged when compared to the control group (Figure 3E–I). Together, these data suggested that PFOS exposure increased the levels of O‐GlcNAcylation mainly by reducing OGA to inhibit removing the GlcNAc moiety from proteins.

### 2.4 PFOS Promoted PES1 Expression by Increasing the Level of FOXK1 O‐GlcNAcylation at Thr573

**Figure 4:**
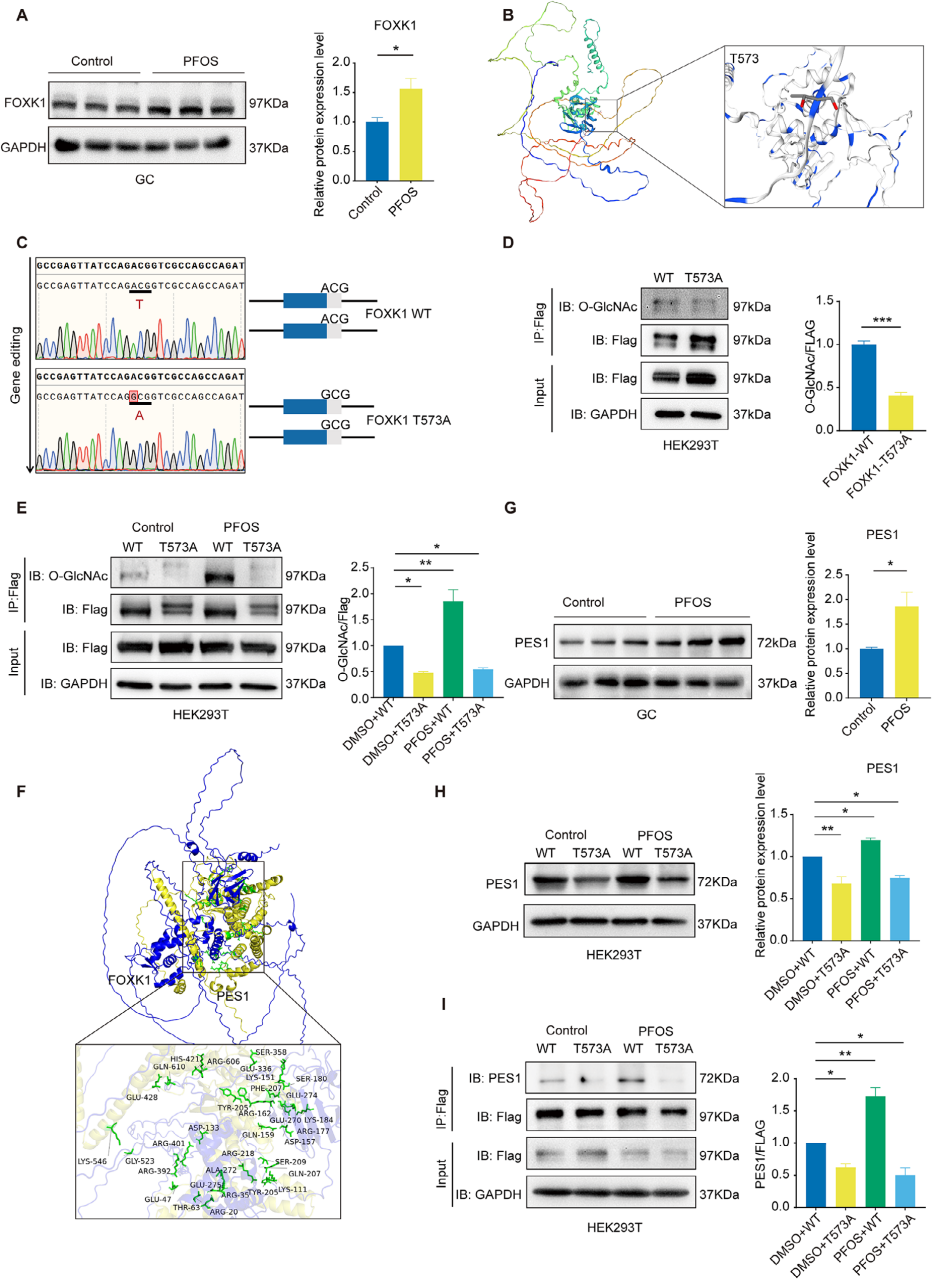
PFOS promoted PES1 expression by increasing the level of FOXK1 O‐GlcNAcylation at Thr573. A) Relative protein expression levels of FOXK1 in GCs exposed to PFOS (*n* = 3). B) 3D structure of the FOXK1 protein. C) Schematic of gene editing. D) FOXK1‐WT and FOXK1‐Thr573 mutant plasmids were overexpressed in HEK293T cells. Immunoprecipitation of FLAG‐FOXK1 was performed with FLAG beads, followed by immunoblotting analysis using O‐GlcNAcylation antibodies (*n* = 3 independent experiments). E) PFOS treatment of HEK293T cells was accompanied by overexpression of FOXK1‐WT and FOXK1‐Thr573 mutant plasmids. Immunoprecipitation of FLAG‐FOXK1 was performed with FLAG beads, followed by immunoblotting analyses using O‐GlcNAcylation antibodies (*n* = 3 independent experiments). F) The 3D binding mode of FOXK1 with PES1 (Blue: FOXK1; Yellow: PES1). G) Relative protein expression of PES1 in GCs exposed to PFOS (*n* = 3). H) Immunoblot analysis of PES1 protein expression in PFOS‐treated HEK293T cells co‐expressing FOXK1‐WT and FOXK1‐Thr573 mutant plasmids (*n* = 3 independent experiments). I) Analysis of FOXK1‐PES1 binding interactions. HEK293T cells overexpressing FLAG‐tagged FOXK1‐WT or FOXK1‐Thr573 mutant plasmids were treated with PFOS, followed by FLAG immunoprecipitation and immunoblotting with an anti‐PES1 antibody (*n* = 3 independent experiments). Statistics: Data are represented as mean ± SEM. *P* values were determined by one‐way ANOVA with Dunnett's multiple comparisons test and two‐tailed unpaired *t*‐tests. ^*^ *P* < 0.05; ^**^ *P* < 0.01.

Among 7 differentially O‐GlcNAcylation proteins, the protein interaction network analysis revealed host cell factor C1 (HCFC1) interacted with forkhead box k1 (FOXK1) (Figure S4A, Supporting Information). Protein expression analysis showed that PFOS exposure significantly increased FOXK1 level (**Figure** **4**A) and did not alter HCFC1 level in GCs (Figure S4B,C, Supporting Information). FOXK1 and its threonine (Thr) 573 O‐GlcNAcylation site was thus subsequently selected for further mechanistic exploration. Subsequently, we used the SWISS model (https://www.swissmodel.expasy.org/) to predict the 3D structure of the FOXK1 protein (Figure 4B). We designed and constructed a plasmid platform to mutate FOXK1 threonine to alanine (Ala), rendering it incapable of O‐GlcNAcylation (Figure 4C). The expression efficiency of the plasmid was verified, and suggested the wild type (WT) and mutation plasmids were in work (Figure S4D–F, Supporting Information). Upon Thr573 mutation, O‐GlcNAcylation of FOXK1 was decreased dramatically (Figure 4D). Next, we also used HEK293T cell line model to investigate O‐GlcNAcylation levels when PFOS exposure treatment transfected with WT (FOXK1 WT) or mutant plasmids (FOXK1 T573A), respectively. PFOS exposure increased the levels of O‐GlcNAcylation while decreased undergoing transfected with mutant plasmids in PFOS exposure group (Figure 4E). Taken together, PFOS exposure increased the O‐GlcNAcylation level of FOXK1 at Thr573 site.

To investigate how FOXK1 O‐GlcNAcylation functions in sex hormone regulation, we applied Co‐IP and LC‐MS/MS analysis to identify its bound proteins on HEK293T cell line model. A total of 16 differentially bound proteins were enriched between the WT and Thr573 mutant groups with PFOS treatment (Figure S4G, Supporting Information). PES1 was reported to mediate estrogen signaling and play an important role in ovarian cancer by regulating ERα and ERβ expression.^[^ ^37^, ^38^ ^]^ Thus, we hypothesized that PES1 was identified as a potential binding protein for FOXK1. Structural models were generated using AlphaFold3, and molecular docking analysis revealed that FOXK1 interacts with PES1 (Figure 4F). We next investigated the protein level of PES1. We observed that PES1 expression was significantly increased in PFOS‐treated GCs (Figure 4G). In addition, PES1 expression was up‐regulated in PFOS‐treated HEK293T cells, but Thr573 mutant suppressed the increased level of PES1 caused by PFOS exposure when compared to PFOS and WT plasmid treatments (Figure 4H). Consistent with protein expression levels, the FOXK1 Thr573 mutant inhibited the PFOS‐induced the increasing binding between PES1 and FOXK1 (Figure 4I). Together, PFOS exposure elevated FOXK1 binding ability to PES1 and its protein level by increasing the level of O‐GlcNAcylation of FOXK1 at Thr573.

### 2.5 PFOS Exposure Inhibited Degradation of PES1 by the FOXK1 Thr573 O‐GlcNAcylation Mediated Ubiquitin‐Proteasome Pathway

**Figure 5:**
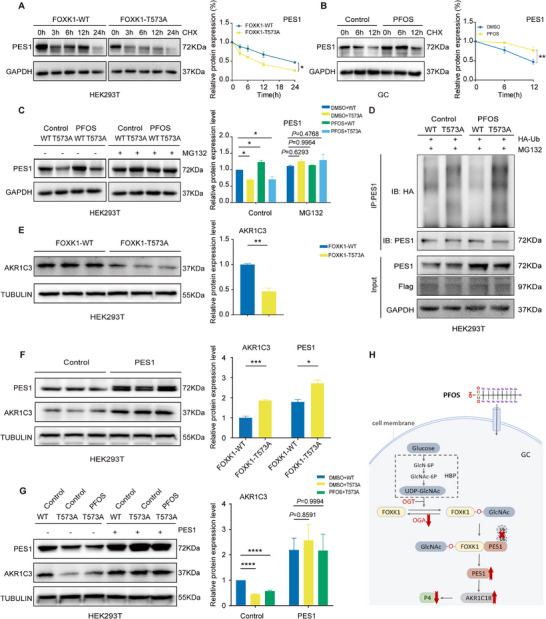
PFOS exposure‐elevated PES1 promoted AKR1C18 to reduce P4 levels. A) HEK293T cells were transfected with FOXK1‐WT or FOXK1‐Thr573 mutant plasmids and treated with cycloheximide (CHX, 50 µg mL^−1^) for specified durations, followed by immunoblot analysis of PES1 (*n* = 3 independent experiments). B. GCs were exposed to PFOS and treated with cycloheximide (CHX, 50 µg mL^−1^) for specified durations, followed by immunoblot analysis of PES1 (*n* = 3 independent experiments). C) HEK293T cells were treated with PFOS and transfected with FOXK1‐WT or FOXK1‐Thr573 mutant plasmids for 48 h, followed by treatment with or without MG132 (25 µM, 6 h) prior to immunoblot analysis of PES1 (*n* = 3 independent experiments). D) Flag‐FOXK1‐WT or FOXK1‐Thr573 mutant plasmids were co‐expressed with HA‐Ub in PFOS‐treated HEK293T cells. After treatment with MG132 (25 µm, 6 h), IP was performed with PES1 antibody, followed by immunoblotting with the indicated antibodies (*n* = 3 independent experiments). E) Relative protein expression levels of AKR1C3 in HEK293T that overexpress FOXK1‐WT or FOXK1‐Thr573 mutant plasmids (*n* = 3). F) Relative protein expression levels of AKR1C3 in HEK293T that overexpressed PES1. G) AKR1C3 protein levels were analyzed by immunoblotting in HEK293T cells following PFOS treatment and transfection with FOXK1‐WT or FOXK1‐Thr573 mutant plasmids, with or without PES1 overexpression (*n* = 3 independent experiments). H) FOXK1 O‐GlcNAcylation‐PES1‐AKR1C18 axis mechanism. Statistics: Data are represented as mean ± SEM. *P* values were determined by one‐way ANOVA with Dunnett's multiple comparisons test, two‐way ANOVA with Bonferroni multiple comparisons test and two‐tailed unpaired *t*‐tests. ^*^ *P* < 0.05; ^**^ *P* < 0.01.

To elucidate how FOXK1 O‐GlcNAcylation regulates PES1, we investigated the ubiquitin‐proteasome pathway of PES1. We evaluated the endogenous levels of PES1 by treating with actinomycinone (CHX; an inhibitor of protein synthesis) in GCs and HEK293T cells. As expected, endogenous PES1 protein expression was decreased after CHX treatment in both FOXK1 WT and FOXK1 Thr573 mutant groups. Moreover, the degradation of PES1 was partly inhibited by PFOS exposure or FOXK1 O‐GlcNAcylation at Thr573 (**Figure** **5**A,B). We also applied MG132 (a protease inhibitor) to treat the HEK293T cells. We found that MG132 treatment partly blocked the degradation of PES1 (Figure 5C). In line with the results, the ubiquitination levels of PES1 were weakened by PFOS and FOXK1 O‐GlcNAcylation at Thr573 in HA‐ubiquitin (HA‐Ub) transfected HEK293T cells (Figure 5D). Taken together, these data suggested that PFOS exposure inhibited PES1 degradation through FOXK1 Thr573 O‐GlcNAcylation mediated ubiquitin‐proteasome pathway, thereby increasing PES1 protein expression level.

### 2.6 PFOS Exposure‐Elevated PES1 Promoted AKR1C18 to Reduce P4 Levels

AKR1C18, which belongs to the aldo‐keto reductase (AKR) subfamily, was reported to regulate the inactivation and conversion of progesterone in the ovary.^[^ ^39^ ^]^ It is recognized that human *Akr1c3* is the ortholog of murine *Akr1c18*.^[^ ^40^ ^]^ Human *Akr1c3* has functional similarity to murine *Akr1c18* and also possesses the regulatory role of reducing progesterone to 20α‐hydroxyprogesterone.^[^ ^41^ ^]^ We assessed the protein expression of AKR1C18 in GCs and AKR1C3 in HEK293T cells, respectively. We found the alterations of AKR1C18 and AKR1C3 protein levels were consistent in both GCs and HEK293T cells (Figures 2J and 5E). To clarify how PES1 regulates AKR1C18, mediating P4 level, we established HEK293T cell line model overexpressed by PES1. Overexpression of PES1 promoted AKR1C3 protein expression in HEK293T cells (Figure 5F). Moreover, overexpression of PES1 rescued the suppression of AKR1C3 expression resulting from the inhibition of FOXK1 O‐GlcNAcylation (via the FOXK1 T573A mutation, Figure 5G). These results indicated that PFOS exposure‐induced PES1 up‐regulation promoted AKR1C18 to reduce P4 production resulting in the inhibition of follicle development, thereby delaying oocyte maturation and early embryonic development.

## 3 Discussion

In this study, we observed that PFOS exposure reduced steroid hormone and impaired oocyte meiosis in a dose‐dependent manner. Moreover, we reported for the first time on a physical interaction between FOXK1 and PES1. Under PFOS stimulation, increased O‐GlcNAcylation of FOXK1 inhibited the ubiquitinated degradation of PES1, thereby upregulating AKR1C18 expression, interfering the ovarian microenvironment and affecting early embryonic development (Figure 5H). Our study elucidates how PFOS indirectly impairs oocyte quality and microenvironment homeostasis by modulating the post‐translational modification status of the key transcription factor FOXK1 in granulosa cells and reveals the novel insights into the female reproductive toxicity of PFOS.

Increasing epidemiological studies indicated that PFOS, widely detected in serum and follicular fluid, may adversely affect oocyte yield, fertilization rates, and high‐quality embryo formation in women undergoing IVF treatment.^[^ ^5^, ^42^ ^]^ GVBD and PBE are key markers of meiotic progression during oocyte IVM.^[^ ^43^ ^]^ PFOS exposure reduced the PBE rate in porcine oocytes,^[^ ^44^ ^]^ which is consistent with our finding. Exposure to 600 µm PFOS disrupted spindle assembly and chromosome alignment.^[^ ^13^ ^]^ However, we did not find significant effects of PFOS treatment at human internal exposure levels in both MI and MII‐stage oocytes. A previous study showed that when bovine oocytes were exposed to 53 ng g^−1^ PFOS for 44 h, the likelihood of cleavage to the two‐cell stage was reduced. Consistent with our results, PFOS may have delayed the timing of the first cleavage division.^[^ ^45^ ^]^ We did not observe a decrease in later embryonic development rates, suggesting that this effect may be due to a delay in developmental progression rather than an increase in early embryonic lethality. Two‐cell stage is the critical period for zygotic genome activation.^[^ ^46^, ^47^ ^]^ Despite the absence of significant effects on morula or blastocyst formation rates, the transient developmental delay induced by PFOS might disrupt zygotic genome activation, with potential long‐term repercussions for the embryo.

FOXK1 has been reported to inhibit GC proliferation and disrupt follicular and oocyte development in rats.^[^ ^48^ ^]^ We also found PFOS exposure induced the binding level of PES1 and FOXK1. Additionally, the FOXK1 Thr573 mutant inhibited the PFOS‐induced binding of PES1 and FOXK1, suggesting the vital role of FOXK1 in this study.

In this study, we observed elevated FOXK1 O‐GlcNAcylation in both PFOS‐exposed ovarian and GCs. HBP provides UDP‐GlcNAc for protein O‐GlcNAcylation.^[^ ^49^ ^]^ OGT facilitates the transfer of GlcNAc moieties from UDP‐GlcNAc to the serine or threonine sites of target proteins. In contrast, OGA reverses this post‐translational modification through its hydrolytic activity. To investigate the mechanism underlying increased FOXK1 O‐GlcNAcylation, we analyzed the expression of key genes involved in UDP‐GlcNAc biosynthesis and no significant alterations were observed. Emerging evidence suggested that O‐GlcNAcylation can also be regulated through nutrient‐independent mechanisms.^[^ ^50^ ^]^ We observed a significant reduction of OGA. In amyotrophic lateral sclerosis (ALS), the oxidative stress signaling molecule NPGPx has been shown to enhance cellular adaptation to oxidative stress by inhibiting OGA and consequently elevating O‐GlcNAcylation levels.^[^ ^51^ ^]^ In addition, PFOS exacerbated oxidative stress and inflammation through activation of the TLR4/MyD88/NF‐κB signaling pathway, ultimately triggering mitochondrial apoptosis.^[^ ^52^ ^]^ Building upon these findings, we propose that PFOS may induce reactive oxygen species (ROS) generation, and that the resulting oxidative stress could specifically inhibit OGA, thereby leading to sustained O‐GlcNAcylation on target proteins.

O‐GlcNAcylation can coat target proteins (whether folded or unfolded, mature or nascent) to prevent aberrant protein aggregation or unwanted modifications. Furthermore, it serves as a molecular “glue” that dynamically regulates protein‐protein interactions in both temporal and spatial dimensions in response to internal and external cues, thereby modulating diverse protein functions within the cell.^[^ ^53^ ^]^ Consistently, we demonstrated that O‐GlcNAcylation on FOXK1 prevented PES1 degradation by inhibiting its ubiquitination. Intriguingly, our data demonstrated that PFOS exposure not only upregulated PES1 expression but also enhanced its physical interaction with FOXK1. A previous study showed that PES1 could be modified by the small ubiquitin‐like modifier (SUMO). One major site (K517) was identified in the C‐terminal Glu‐rich domain of PES1.^[^ ^54^ ^]^ The FOXK1‐PES1 protein complex may sterically occlude ubiquitination sites on PES1, thereby impeding recognition by E3 ubiquitin ligases and subsequently prolonging its half‐life through inhibition of proteasomal degradation.

The end product of HBP pathway, UDP‐GlcNAc, served as a substrate for both N‐glycosylation and O‐glycosylation,^[^ ^55^ ^]^ which indicated that N‐glycosylation may potentially be involved in PFOS‐induced biological responses. OGT can directly catalyze the transfer of UDP‐GlcNAc to serine/threonine hydroxyl groups of target proteins via β‐linkage, forming dynamic and reversible O‐GlcNAcylation. However, UDP‐GlcNAc is an indirect donor for N‐glycosylation, initiating the generation of oligosaccharide precursors on the cytoplasmic side of the endoplasmic reticulum (ER).^[^ ^56^ ^]^ Studies reported that inhibition of OGT led to aberrant actin and microtubule organization in oocytes.^[^ ^25^, ^57^ ^]^ Therefore, we primarily elucidated the functional role of O‐GlcNAcylation in this biological context.

PES1 is a nucleolar protein that plays critical roles in embryonic development and ribosome biogenesis.^[^ ^58^ ^]^ Our study revealed that PES1 is upregulated in granulosa cells upon PFOS exposure. Previous studies have demonstrated that reduced PES1 protein levels impair its binding to target gene promoters, leading to decreased expression of p300 and Caspase1, thereby disrupting p300‐mediated acetylation of SREBP1c and inflammatory responses.^[^ ^59^ ^]^ Based on these findings, we hypothesized that PES1 might function as a transcriptional coactivator or directly bind to the promoter region of AKR1C18 to regulate its transcription, consequently influencing progesterone levels.

PFOS affects oocyte quality and subsequent embryonic development through multiple mechanisms. In oocyte, PFOS disrupted key processes, including cytoskeletal dynamics and meiotic progression.^[^ ^13^ ^]^ PFOS also promoted oxidative stress by disrupting tryptophan metabolism and inducing ferroptosis.^[^ ^60^, ^61^ ^]^ These findings highlight a critical impact on functional integrity of the oocyte.

Previous studies demonstrated that PFAS mixtures inhibited steroid secretion in ovarian GCs, thereby impairing follicular development.^[^ ^20^, ^62^ ^]^ In our study, we observed that PFOS exposure reduced both E2 and P4. Cytochrome P450scc (CYP11A1), Cytochrome P450c17 (CYP17A1), and 3β‐hydroxysteroid dehydrogenase (3β‐HSD) are all key enzymes in the steroid hormone biosynthesis pathway.^[^ ^63^ ^]^ However, PFOS exposure only significantly affected AKR1C18 expression. AKR1C18, belonging to the aldo‐keto reductase (AKR) superfamily, catalyzed the inactivation of progesterone in the ovary. Additionally, it modulated the binding of progestins to progesterone receptors, playing a significant role in ovarian function.^[^ ^39^ ^]^ Our findings suggested that PFOS might disrupt progesterone metabolism by influencing AKR1C18 expression, contributing to the observed decline in P4. P4 provides antioxidant defence and boosts mitochondrial potential.^[^ ^64^, ^65^ ^]^ Its loss may cause oxidative stress and mitochondrial failure, potentially impairing oocyte competence.^[^ ^66^ ^]^ LH surge is a critical event that triggers complete reprogramming of follicular function.^[^ ^67^ ^]^ Insufficient P4 may delay the LH surge, thereby impairing oocyte maturation.^[^ ^68^ ^]^ Compromised oocyte developmental potential will adversely affect early embryonic development.

Our study has several limitations. First, while our findings highlighted the significance of the FOXK1/PES1 axis, the potential involvement of other significantly differential O‐GlcNAcylated proteins need further investigation. Second, although we observed a strong inverse correlation between AKR1C18 expression and P4, direct evidence that AKR1C18 is responsible for the PFOS‐induced reduction in P4 is still lacking. Future studies were warranted to address these limitations.

## 4 Conclusion

In summary, this study demonstrated that PFOS exposure delayed oocyte and early embryonic development. Mechanically, O‐GlcNAcylation of FOXK1 at Thr573 triggered a downstream network involving PES1 and AKR1C18, thereby perturbing progesterone metabolism and contributing to the observed developmental impairments. Our study reveals a novel mechanism and provides new insights into the reproduction toxicity of PFOS.

## 5 Experimental Section

### Mice and PFOS Treatment

All studies involving animals were approved by the Institutional Animal Care and Use Committee of Nanjing Medical University (IACUC‐2107010). Female C57BL/6J mice (3–5 weeks old) were obtained from the Production Department of Nanjing Medical University and maintained under standard conditions (12 h light/dark cycle, 23 ± 1 °C).

The species‐specific equivalent dose was calculated using the following formula:

(1) $$\mathrm{Animal}\;\mathrm{Dose}=\mathrm{Human}\;\mathrm{Dose}\times \left(\mathrm{Human}\;K_{m}/\mathrm{Animal}\;K_{m}\right)$$

*K*_m_ (allometric scaling factor, typically defined as body weight (kg) divided by body surface area (m^2^)).

Based on reported geometric mean serum PFOS levels (8–10 µg L^−1^) in women,^[^ ^69^, ^70^ ^]^ the internal exposure dose in humans was calculated as 0.9091 ng kg^−1^ (an average human body weight of 55 kg and blood volume of 5L is assumed). The equivalent dose of PFOS in mice was 0.0091 mg kg^−1^, and it was obtained by multiplying the dose in humans by the body surface area conversion factor (12.3). (Approximately equal to 10).^[^ ^71^ ^]^ Ultimately, the internal human exposure dose was established at 0.01 mg kg^−1^ day^−1^, with three experimental dosage groups set at 0.001, 0.01, and 1 mg kg^−1^ day^−1^, respectively.

PFOS (90% purity) (CAS: 1763‐23‐1) was obtained from Laiyao Biotechnology Ltd (Xinyang, China). Working stocks were diluted with 0.001% DMSO (CAS: 67‐68‐5, Sigma–Aldrich, USA) in PBS. For in vivo exposure, 40 wild‐type mice were randomly divided into four treatment groups. All animals received daily oral gavage of PFOS at different doses (0, 0.001, 0.01, and 1 mg kg^−1^ day^−1^) for two weeks. PFOS was administered at concentrations of 0.02, 0.2, and 20 µM for in vitro exposure.

### Cell Culture and Treatment

HEK293T cells (CRL‐11268, ATCC, USA, RRID: CVCL_1926) and GC cells (Delf‐16320, Hefei Wanwu Biotechnology, China, RRID: CVCL_F0QC) were maintained in DMEM (PM150210, Procell, China) or DMEM F12 (PM150315, Procell, China) containing 10% fetal bovine serum (164210‐50, Procell, China) and 1% penicillin/streptomycin (BL505A, Biosharp, China). All cell cultures tested negative for mycoplasma. To validate the purity of the GC line, immunofluorescence staining was applied and found 98.68% of GCs were positively marked with follicle‐stimulating hormone receptor (FSHR) (Figure S2A, Supporting Information). Cell transfection was performed using Lipofectamine 3000 (L3000075, ThermoFisher, USA) according to the manufacturer' s protocol. For exogeneous expression, HEK293T cells were transfected with plasmids carrying pCMV3‐SV40‐EGFP(2A)Puro‐c3×Flag‐M‐Foxk1, pCMV3‐SV40‐EGFP(2A)Puro‐c3 × Flag‐M‐Foxk1 (T573A) and pCMV3‐SV40‐EGFP(2A)Puro‐NC.

### Immunofluorescence (IF)

Following IVM, oocytes were fixed in 4% paraformaldehyde (30 min), permeabilized with 0.5% Triton X‐100 (20 min), and blocked with 1% BSA (1 h, RT). Primary antibody incubation was performed overnight at 4 °C, followed by 2 h incubation with secondary antibodies at RT. DAPI (P36941, Invitrogen, USA) was added to stain nuclei. Finally, oocytes were observed under Confocal Microscope LSM700 (Carl Zeiss AG).

### Hematoxylin‐Eosin Staining

The ovaries were collected and fixed in 4% paraformaldehyde for 24 h, and embedded in paraffin after dehydration in different concentrations of alcohol. Tissue samples were sectioned at 5 µm and stained with hematoxylin and eosin (H&E). Digital images of histological sections were acquired with a Pannoramic MIDI scanner (3DHISTECH, Hungary) at objective magnifications of 200× and 400×. Follicle counts were performed on ovarian sections for each group. Only follicles containing a nucleated oocyte were counted. Follicles surrounded by flat granulosa cells were classified as primordial follicles. Follicles with a granulosa cell layer of 5 to 6 layers surrounding the oocyte were classified as primary follicles. Follicles with emerging antral spaces were identified as secondary follicles. Secondary follicles continued to develop to the final stage, volume of follicular fluid in the follicle cavity increased and protruded to the surface of the ovary to become mature follicles.

### ELISA Assay

Serum samples were collected from PFOS‐exposed mice, and culture supernatants were obtained from GCs following 48 h PFOS exposure with subsequent 24 h incubation in medium containing 10 nmol L^−1^ testosterone. E2 and P4 levels in the samples were measured using ELISA kits (BPE20376 and BPE20381, Shanghai Lengton Bioscience, China) following the manufacturer's protocol. The samples and detection reagents were incubated in a 96‐well plate at 37 °C for 30 min. After washing, the chromogenic solution was added and incubated at 37 °C for 10 min in the dark. Absorbance (OD450) was then measured using a SpectraMax Absorbance Reader (Molecular Devices, Sunnyvale, USA).

### O‐GlcNAcylated Peptides Enrichment and Identification

Proteomics analysis of O‐GlcNAcylation was provided by Jingjie PTM BioLab (China). Briefly, ovarian tissues from DMSO control and 1 mg kg^−1^ day^−1^ PFOS‐exposed groups were lysed for protein extraction. Equal protein amounts were subjected to tryptic digestion, followed by enrichment of modified peptides. The samples were then analyzed using label‐free quantitative proteomics via liquid chromatography‐tandem mass spectrometry (LC‐MS/MS). By comparing signal intensities of corresponding peptides across samples, relative protein quantification was achieved.

### Western Blotting Analysis

Total protein was extracted using RIPA Lysis Buffer (P0013B, Beyotime Biotech, China) with PMSF (ST506, Beyotime Biotech, China). Proteins (20–40 µg) were separated by SDS polyacrylamide gels (SDS‐PAGE) and immobilized on PVDF membranes (1 620 264, Bio‐Rad, USA). Membranes were incubated with primary antibodies overnight at 4 °C and further incubated with horseradish peroxidase (HRP)‐conjugated secondary antibodies. Protein signals were detected by using enhanced chemoluminescence regent (ECL, E411‐04, Vazyme, China). β‐Tubulin and GAPDH served as internal control. Image J (v. 1.8.0) was used for quantified intensity of bands. Band intensities were normalized to internal controls and subsequently to the mean of the control group, and data are expressed as relative expression levels (mean ± SEM). Information on antibodies is given in Table S3 (Supporting Information).

### Co‐Immunoprecipitation (Co‐IP) Assay

Cell lysis was performed using RIPA buffer (Beyotime Biotech) containing PMSF. Following overnight incubation with primary antibodies or rabbit IgG at 4 °C, Protein A/G agarose beads (Sigma–Aldrich) were added and incubated for 2–4 h. Protein samples were then resolved by SDS‐PAGE and analyzed through Western blotting.

### Quantitative RT‐PCR Analysis

Total RNA was extracted using the FreeZol Reagent kit (R711‐01, Vazyme, China). RNA concentration was measured with NanoDrop 2000 spectrophotometer (Thermo Fisher Scientific, USA). The extracted RNA were converted into cDNA by HiScript III RT SuperMix for qPCR (R323‐01, Vazyme, China). Quantitative RT‐PCR was implemented by ChamQ Universal SYBR qPCR Master Mix (Q711‐02, Vazyme, China). Primer sequences are provided in Table S4 (Supporting Information). Relative quantification was achieved by normalizing to the reference gene *Gapdh*, and the fold change was calculated via the 2^(‐ΔΔCT) method.

### Statistical Analysis

Statistical analysis was performed using GraphPad Prism (v9.0) and RStudio. Data were analyzed by one‐way ANOVA or Two‐way ANOVA followed by Dunnett's, Tukey's or Bonferroni multiple comparisons test. Unpaired two‐tailed *t*‐test for comparisons between two independent groups only (alpha = 0.05). All data are presented as mean ± SEM. Significant differences between treatment and control groups were denoted as ^*^ *P* < 0.05, ^**^ *P* < 0.01, ^***^ *P* < 0.001 and ^****^P < 0.0001.

## Conflict of Interest

The authors declare no conflict of interest.

## Author Contributions

S.H., Q.Y., Z.W., and Y.F. contributed equally to this work. S.H., Q.Y., Z.W., and Y.F. conducted the functional experiments, prepared the figures and wrote the original manuscript. H.Q., J.Z., Y.Z., and K.D. prepared the figures and assisted in the performance of the experiments. L.S., H.X., H.S., and Y.G. conducted animal experiments. G.D. and D.W. provided materials and supervision. Y.F. and C.L. provided funding acquisition and designed the experiments.

## Supporting information



Supporting Information

Supplemental Table1

Supplemental Table2

Supplemental Table3

Supplemental Table4

## Data Availability

The data that support the findings of this study are available on request from the corresponding author. The data are not publicly available due to privacy or ethical restrictions.
