# Supplementary material for: PFOS Disrupts Oocyte Maturation and Early Embryonic Development via Ovarian FOXK1 O‐GlcNAcylation in Mice
Source: Adv Sci (Weinh). 2025 Dec 12;13(12):e14857. doi: 10.1002/advs.202514857 (PMC12948240; doi:10.1002/advs.202514857)
Supplement: Supplementary file 1 — Supporting Information [file ADVS-13-e14857-s005.docx]

Supporting Information

**Title**: PFOS Disrupts Oocyte Maturation and Early Embryonic Development via Ovarian FOXK1 O-GlcNAcylation in Mice

Shuwen Han ^a, b, 1^, Qin Yuan ^a, b, 1^, Zhu Wu ^a, b, 1^, Yaohui Fang ^a, b, 1^, Hong Qian ^a, b^, Jiale Zhu ^a, b^, Yuchen Zhang ^a, b^, Ke Deng ^a, b^, Liangliang Su ^a, b^, Haibo Xu ^a, b^, Haotian Shu ^a, b^, Yiming Gong ^a, b^, Qiaoqiao Xu ^a, b^, Guizhen Du ^a, b^, Di Wu ^a, b^, Yun Fan ^a, b, c, *^, Chuncheng Lu ^a, b, *^

^a^ State Key Laboratory of Reproductive Medicine, Center for Global Health, School of Public Health, Nanjing Medical University, Nanjing 211166, China

^b^ Key Laboratory of Modern Toxicology of Ministry of Education, School of Public Health, Nanjing Medical University, Nanjing 211166, China

^c^ Department of Microbes and Infection, School of Public Health, Nanjing Medical University, Nanjing 211166, China

^1^ The first four authors contributed equally to this work.

^*^ To whom correspondence should be addressed at:

Dr. Chuncheng Lu and Dr. Yun Fan

State Key Laboratory of Reproductive Medicine, Center for Global Health, Nanjing Medical University, Nanjing 211166, China

Tel: +86-25-86868420 Fax: +86-25-86862847

Email: [chunchenglu@njmu.edu.cn](mailto:chunchenglu@njmu.edu.cn), yunfan@njmu.edu.cn


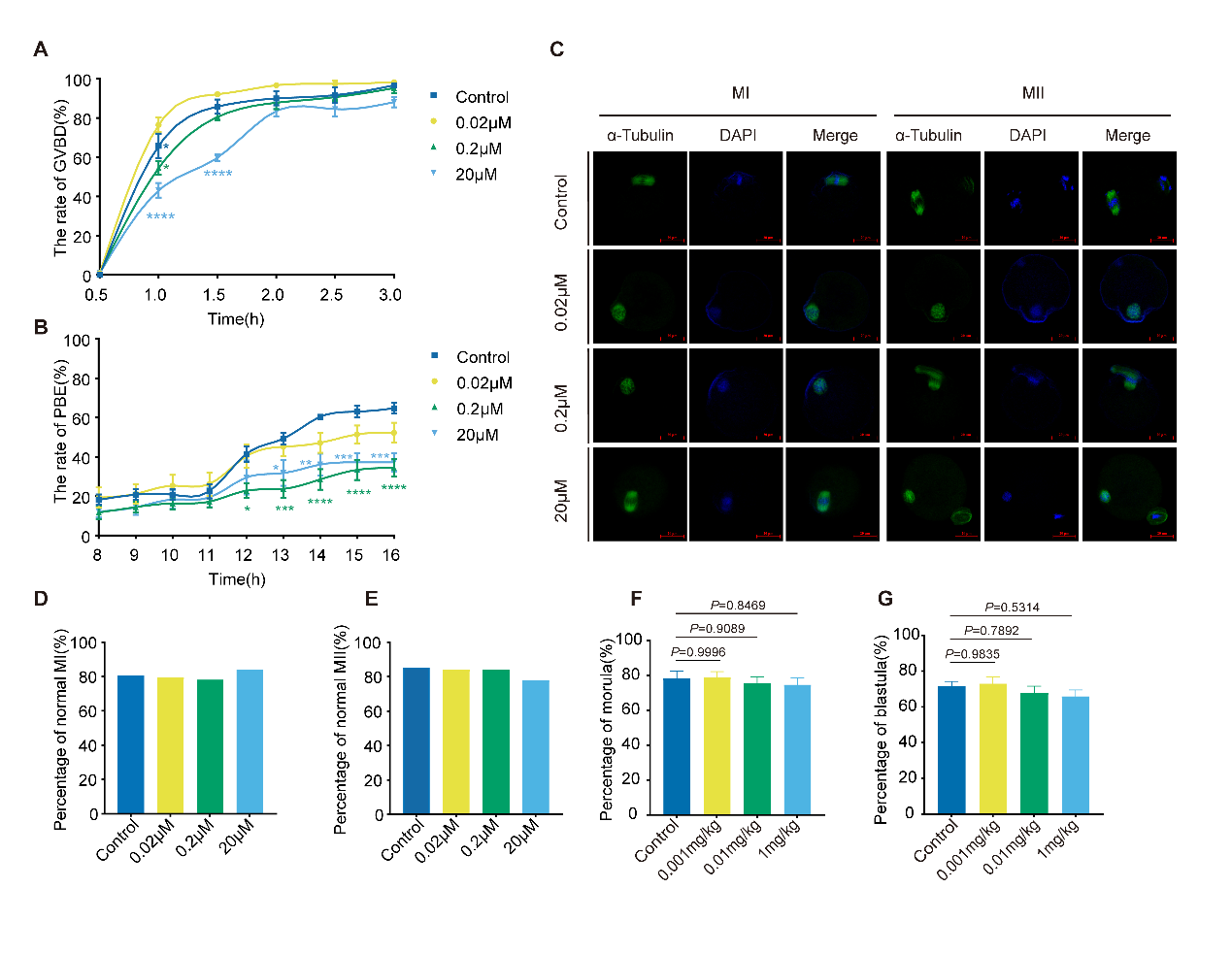


Figure S1. Effects of PFOS exposure.

A. GVBD rate in oocytes after PFOS treatment (Control, n=120; 0.02µM, n=116; 0.2µM, n=108; 20µM, n=109). B. PBE rate in oocytes after PFOS treatment (Control, n=120; 0.02µM, n=116; 0.2µM, n=108; 20µM, n=109). C. Immunofluorescence images of spindles and chromosomes in MI- and MII-stage oocytes after PFOS treatment (after 16h in vitro maturation; Bar = 20 µm). D. Proportion of MI oocytes with normal spindle/chromosome alignment (Control, n=31; 0.02μM, n=34; 0.2μM, n=64; 20μM, n=57). E. Proportion of MII oocytes with normal spindle/chromosome alignment (Control, n=62; 0.02μM, n=50; 0.2μM, n=19; 20μM, n=27). F. The rate of development of morula (72h after fertilization; Control, n=297; 0.02µM, n=287; 0.2µM, n=374; 20µM, n=360). G. The rate of development of blastula (96h after fertilization; Control, n=297; 0.02µM, n=287; 0.2µM, n=374; 20µM, n=360). Statistics: Data are represented as mean ± SEM. *P* values were determined by one-way ANOVA or two-way ANOVA followed by Dunnett' s or Tukey' s multiple comparisons test. **P* < 0.05; ***P* < 0.01; ****P*< 0.001; *****P* < 0.0001.


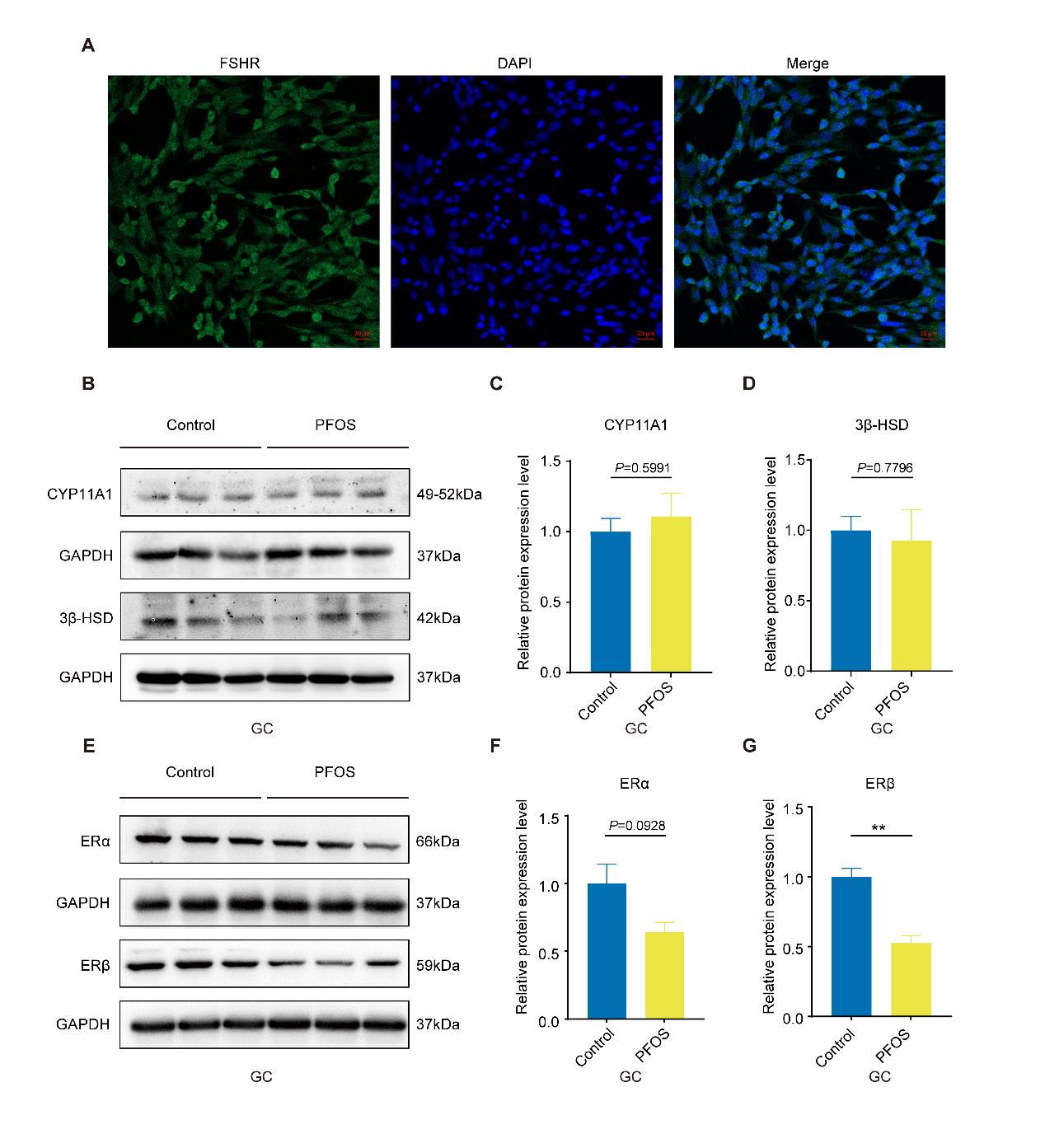


Figure S2 Effects of PFOS exposure on hormone receptors and enzymes in steroid hormone synthesis.

A. Immunofluorescence staining of FSHR in granulosa cells (Bar = 20 µm). B-D. Relative protein expression level of CYP11A1, 3β-HSD in GCs exposed to PFOS (n=3). E-G. Relative protein expression level of ERα, ERβ in GCs exposed to PFOS (n=3). Statistics: Data are represented as mean ± SEM. *P* values were determined by two-tailed unpaired t-tests. **P* < 0.05; ***P* < 0.01.


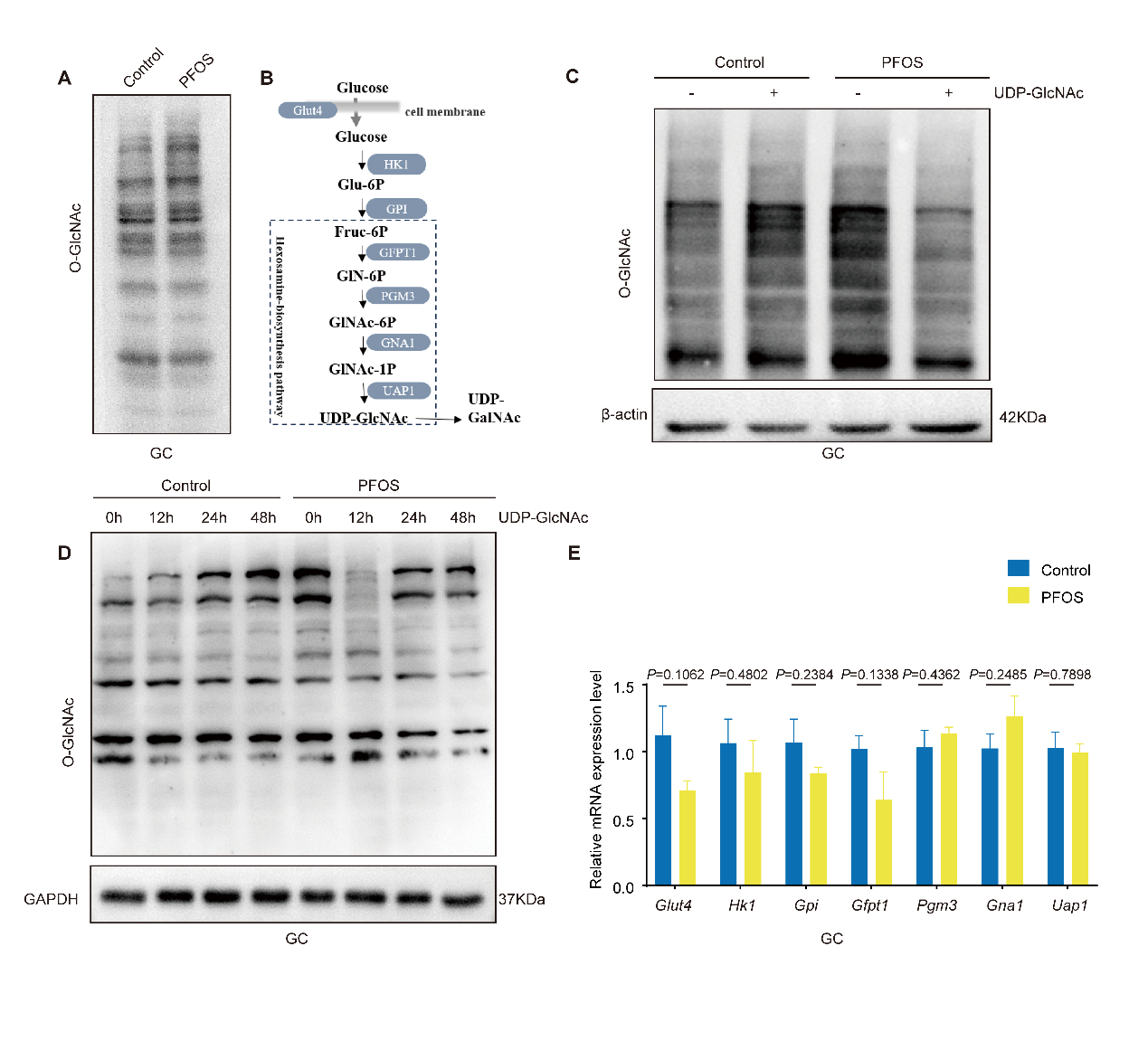


Figure S3. The expression of UDP-GlcNAc and key genes involved in UDP-GlcNAc biosynthesis.

A. Total O-GlcNAcylation levels in GCs exposed to PFOS. B. Schematic diagram of the UDP-GlcNAc generation process (n=3 independent experiments). C. Total O-GlcNAcylation levels in GCs exposed to PFOS and treated UDP-GlcNAc for 48 h (n=3 independent experiments). D. Total O-GlcNAcylation levels in GCs exposed to PFOS and treated UDP-GlcNAc for 12, 24 and 48h (n=3 independent experiments). E. Gene expression levels of *Glut4*, *Hk1*, *Gpi*, *Gfpt1*, *Pgm3*, *Gna1*, and *Uap1* during UDP-GlcNAc generation process (n=5-6). Statistics: Data are represented as mean ± SEM. *P* values were determined by two-tailed unpaired t-tests.


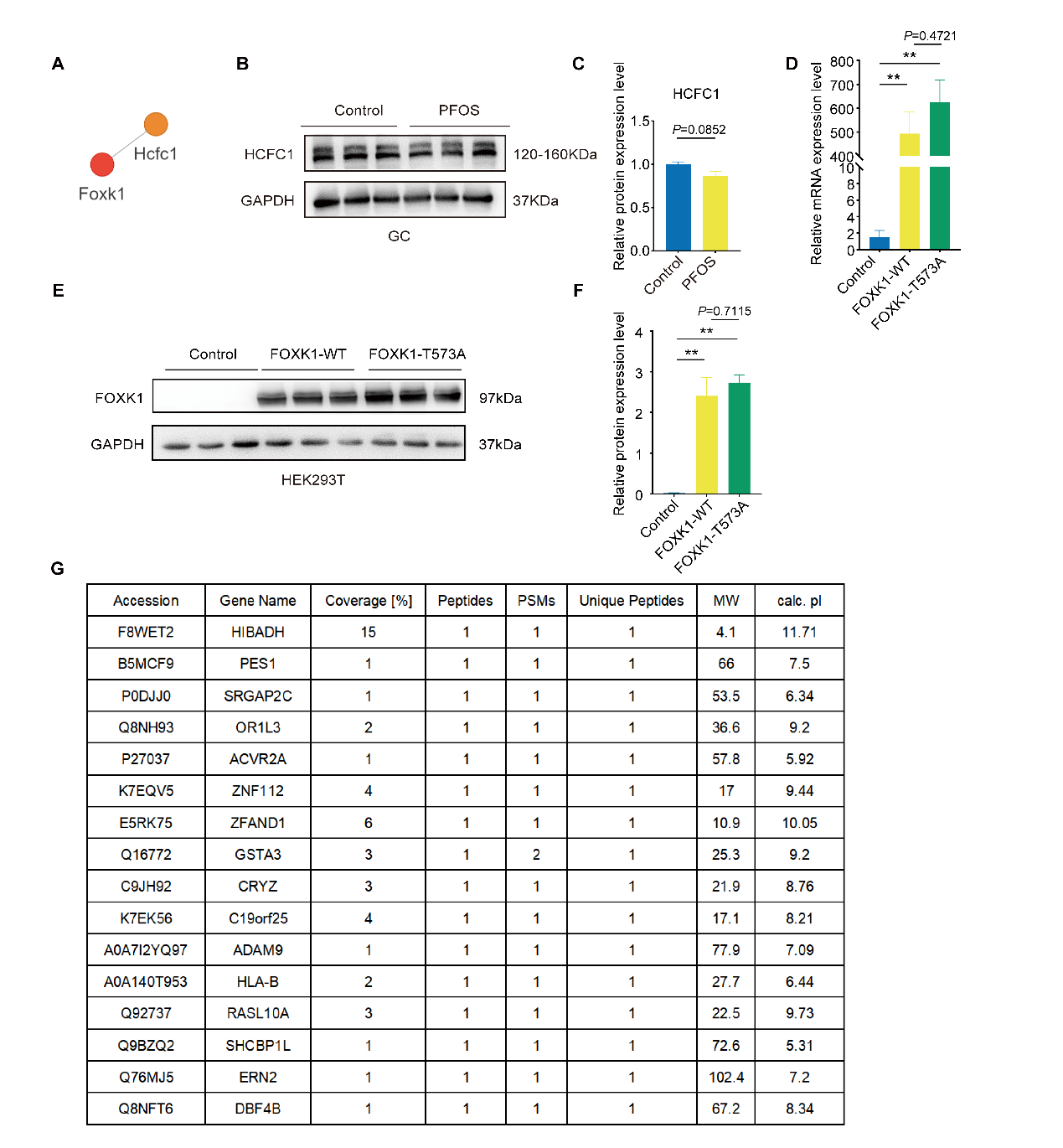


Figure S4. FOXK1 expression efficiency validation and a catalog of proteins that interact with FOXK1.

A. Host cell factor C1 (HCFC1) was interacted with forkhead boxk1 (FOXK1). B-C. Relative protein expression levels of HCFC1 in GCs exposed to PFOS (n=3). D. Gene expression levels of FOXK1 in HEK293T cells transfected with the FOXK1 WT and T573A plasmids (n=3). E-F. Relative protein expression of FOXK1 in HEK293T cells after transfected with the FOXK1 WT and T573A plasmids (n=3). G. List of FOXK1 differentially binding proteins between the WT and T573A groups in HEK293T with PFOS treatment. Statistics: Data are represented as mean ± SEM. *P* values were determined by one-way ANOVA with Tukey' s multiple comparisons test and two-tailed unpaired t-tests. ***P* < 0.01.
